# Investigating the Gender Differences in Children and Adolescents with Autism Spectrum Disorder: A Literature Review

**DOI:** 10.1192/bjo.2025.10138

**Published:** 2025-06-20

**Authors:** Samantha Ellis, Declan Hyland

**Affiliations:** ^1^School of Medicine, University of Liverpool, Liverpool, United Kingdom; ^2^Mersey Care NHS Foundation Trust, Liverpool, United Kingdom

## Abstract

**Aims:** It is estimated that 1 in 100 individuals in the UK population are on the autistic spectrum. There is known to be a marked gender difference, with a male to female ratio of about 3:1. Previously, this diagnostic discrepancy was attributed to autism spectrum disorder (ASD) being known as a “male-dominant” condition, but in recent years this concept has been disproven and the prevalence of ASD in females is thought to be similar to that of males. This literature review aims to explore current research investigating the gender differences in the clinical presentation of ASD, and how this may contribute to the challenges many girls and women experience in receiving a timely diagnosis.

**Methods:** Three databases (PubMed, Scopus, and Medline) were searched for studies on the gender differences in the presentation of ASD in children and adolescents. In the screening process, an exclusion criterion was applied to the records according to relevance to the research question. The studies identified were then assessed for eligibility against a final inclusion criterion, resulting in six studies being included in the final review.

**Results:** Results showed that girls with ASD typically received a diagnosis at an older age than their male counterparts. Girls were also more likely to receive an alternative diagnosis to ASD, often mental health-related, before being given an ASD diagnosis. Repeated and Restricted Behaviours and Interests (RRBIs), which are considered as characteristic and often diagnostic features in ASD, were more prevalent in boys in early childhood. Girls with ASD often had to present with more severe deficits in intellectual ability (IQ), social and communicative skills, or speech and language skills, to be diagnosed with ASD in comparison with their male counterparts.

**Conclusion:** The studies included in this literature review demonstrated that, whilst in some cases differences in clinical presentation of ASD in boys and in girls may be mild, there are undoubtedly differences present. This is not surprising considering the heterogeneity of ASD as a spectrum disorder. Research investigating the various phenotypes of ASD is hugely beneficial in widening the perception from the traditional concept of ASD being viewed as a “male disorder”. There is a clear need for more research to be done to close the gender gap in the diagnosis of ASD and facilitate more timely diagnosis in girls and women and appropriate interventions at an earlier stage, thereby improving quality of life.

